# Low Completeness of Bacteraemia Registration in the Danish National Patient Registry

**DOI:** 10.1371/journal.pone.0131682

**Published:** 2015-06-29

**Authors:** Kim Oren Gradel, Stig Lønberg Nielsen, Court Pedersen, Jenny Dahl Knudsen, Christian Østergaard, Magnus Arpi, Thøger Gorm Jensen, Hans Jørn Kolmos, Mette Søgaard, Annmarie Touborg Lassen, Henrik Carl Schønheyder, Danish Collaborative Bacteraemia Network (DACOBAN), Danish Observational Registry of Infectious Syndromes (DORIS)

**Affiliations:** 1 Center for Clinical Epidemiology, South, Odense University Hospital, Odense, Denmark; 2 Research Unit of Clinical Epidemiology, Institute of Clinical Research, University of Southern Denmark, Odense, Denmark; 3 Department of Infectious Diseases, Odense University Hospital, Odense, Denmark; 4 Department of Clinical Microbiology, Copenhagen University Hospital, Hvidovre Hospital, Hvidovre, Denmark; 5 Department of Clinical Microbiology, Copenhagen University Hospital, Herlev Hospital, Herlev, Denmark; 6 Department of Clinical Microbiology, Odense University Hospital, Odense, Denmark; 7 Department of Clinical Epidemiology, Institute of Clinical Medicine, Aarhus University Hospital, Aarhus, Denmark; 8 Department of Emergency Medicine, Odense University Hospital, Odense, Denmark; 9 Department of Clinical Microbiology, Aalborg University Hospital, Aalborg, Denmark; 10 Department of Clinical Medicine, Aalborg University, Aalborg Denmark; University College London, UNITED KINGDOM

## Abstract

Bacteraemia is associated with significant morbidity and mortality and timely access to relia-ble information is essential for health care administrators. Therefore, we investigated the complete-ness of bacteraemia registration in the Danish National Patient Registry (DNPR) containing hospital discharge diagnoses and surgical procedures for all non-psychiatric patients. As gold standard we identified bacteraemia patients in three defined areas of Denmark (~2.3 million inhabitants) from 2000 through 2011 by use of blood culture data retrieved from electronic microbiology databases. Diagnoses coded according to the International Classification of Diseases, version 10, and surgical procedure codes were retrieved from the DNPR. The codes were categorized into seven groups, ranked a priori according to the likelihood of bacteraemia. Completeness was analysed by contin-gency tables, for all patients and subgroups. We identified 58,139 bacteraemic episodes in 48,450 patients; 37,740 episodes (64.9%) were covered by one or more discharge diagnoses within the sev-en diagnosis/surgery groups and 18,786 episodes (32.3%) had a code within the highest priority group. Completeness varied substantially according to speciality (from 17.9% for surgical to 36.4% for medical), place of acquisition (from 26.0% for nosocomial to 36.2% for community), and mi-croorganism (from 19.5% for anaerobic Gram-negative bacteria to 36.8% for haemolytic strepto-cocci). The completeness increased from 25.1% in 2000 to 35.1% in 2011. In conclusion, one third of the bacteraemic episodes did not have a relevant diagnosis in the Danish administrative registry recording all non-psychiatric contacts. This source of information should be used cautiously to iden-tify patients with bacteraemia.

## Introduction

Bacteraemia, with its 30-day mortality of 15–30%, is estimated to be one of the top seven causes of death in the developed countries [1]. Bacteraemia is defined as the presence of viable bacteria or fungi in blood cultures (BCs) [2–4]. In contrast, the term sepsis defines a clinical condition reflecting the host response to infection [5]. However, the two terms are often used interchangeably, which may create some confusion.

Regardless of this, both conditions constitute a severe clinical burden which may favour their easy identification and surveillance through administrative hospital discharge registries. Nevertheless, studies have generally indicated high variation and low completeness in administrative registries for both sepsis [6–8] and bacteraemia [9–14].

The Danish National Patient Registry (DNPR) contains hospital discharge diagnoses and surgical procedures from all non-psychiatric inpatients since 1977 [15]. A prior Danish study found that only 18 of 406 (4.4%) bacteraemic episodes in 1994 were recorded with a relevant bacteraemia or sepsis diagnosis in the DNPR [9]. This may have been due to the newly implemented International Classification of Diseases, version 10 (ICD-10) codes in 1994, and the completeness may have improved since then, possibly also encouraged by the Survival Sepsis Campaign [16]. We therefore investigated the completeness of sepsis/bacteraemia ICD-10 codes in the DNPR, using bacteraemic episodes from 2000 through 2011 derived from positive BC data as gold standard. To assess the impact of completeness on prognostic models we applied multivariate regression analyses according to whether the bacteraemic episodes were recorded in the DNPR, using 30-day mortality as outcome.

## Materials and Methods

### Setting

The Danish healthcare system is tax financed and provides care free of charge for all residents. The admission of all acutely ill patients to the nearest public hospital in their area of residence prompts a population-based coverage. Our data covered three geographically well-defined areas (North Denmark Region, Capital Region, Funen County) served by four Departments of Clinical Microbiology (DCMs) in hospitals in Aalborg, Herlev, Hvidovre, and Odense (in total 2.3 million inhabitants [17, 18]). BC procedures have been described previously [19–21].

### Data linkage

All Danish residents have a unique personal identification number used for all health contacts, which permits unambiguous linkage between health administrative registries [22].

### Core dataset

The study periods were 2000–2011 (North Denmark and Capital regions) or 2000–2008 (Funen).

All microbiological results were recorded in an electronic laboratory information system (Aalborg, Herlev, and Hvidovre: ADBakt [Autonik, Sköldinge, Sweden]; Odense: the local Patient Administrative System in 2000–2005 and the MADS system [www.madsonline.dk] thereafter). Key data included dates of draw and receipt of the BC in the DCM, and BC isolates. We retrieved data on all positive BCs and used previously published computer algorithms to exclude likely contaminants and to derive bacteraemic episodes [21, 23]. For each episode we defined the best-estimate baseline date as the date of draw; for bacteraemic episodes with a missing date of draw (9.3%) we used the never-missing date of receipt. We used previously reported computer algorithms to derive incident and non-incident episodes as well as acquisition (community-acquired, healthcare-associated, nosocomial) [21].

### Code systems used in the Danish National Hospital Registry

In Denmark, all diagnostic and surgical procedure codes are allocated by physicians when patients are discharged.

Until 1994, diagnoses were coded according to the International Classification of Diseases, version 8 (ICD-8), and thereafter according to the ICD-10, as ICD-9 was never implemented in Denmark [15]. We used the Danish ICD-10 version [24], derived from the WHO classification, vs. 2010 [25] with amendments that more specifically designate bacteraemia (e.g. A49.9A [Bacteraemia, unspecified] found in the Danish, but not in the WHO, version). For each hospitalization, one obligatory principal diagnosis may be supplemented with up to 20 secondary diagnoses.

For surgical procedures, the Nordic Classification of Surgical Procedures [26] (NOMESCO) has been in use since 1996.

### Retrieval of diagnoses and surgical procedures related to sepsis or bacteraemia

Two authors (HCS, SLN) independently retrieved codes for diagnoses and surgical procedures that may indicate the presence of bacteraemia, either directly by codes using the term bacteraemia or septicaemia or indirectly by codes indicating focal infections. The authors’ codes were combined and consensus was reached with agreement on all included codes, shown in the Appendix.

### Linkage to the Danish National Hospital Registry

We linked the core dataset to their DNPR inpatient data and retrieved the date of hospital admission from home which was closest to and equal to or earlier than the best-estimate baseline date. Likewise, we retrieved the date of discharge to home which was closest to and equal to or later than the best-estimate baseline date. For this hospitalization, which covered the bacteraemic episode, we retrieved all relevant diagnosis and surgical procedure codes (see Appendix).

We then linked the core dataset to the DNPR to retrieve all first-time diagnoses in the Charlson comorbidity index [27] within a 6-year period prior to the best-estimate baseline date. In this index, 19 major disease categories (e.g., malignancy, cardiovascular diseases, and diabetes mellitus) are assigned a score, with higher scores given to prognostically more severe diseases.

### Linkage to the Danish Civil Registration System

To obtain mortality data we linked the study population to the Danish Civil Registration System, which comprises daily updated data on the patients' vital status, as well as date of death, disappearance, or emigration, if relevant [28].

### Statistical analyses

We categorized the codes for diagnoses and surgical procedures into seven groups and determined the following priority list for the likelihood of representing bacteraemia: 1) infections/bacteraemia; 2) other diagnoses/bacteraemia; 3) other diagnoses/focal infection; 4) surgical procedures/focal infection; 5) infections/systemic infection; 6) other diagnoses/systemic infection; 7) infections/focal infection (see Appendix for the specific codes and text examples for the three most common codes within each group). If group 1 occurred in the hospitalization comprising the bacteraemic episode, groups 2–7 were annulled. If group 1 did not occur we retrieved group 2 and annulled groups 3–7. If group 2 did not occur we proceeded to group 3, etc. (see Appendix for examples for two specific bacteraemic episodes).

We used the same seven groups with a different prioritization (5 > 7 > 3 > 4 > 6 > 1 > 2) to derive diagnoses/procedures that may indicate the presence of a focal infection, applying the same principles as for bacteraemia (if 5 occurred, 1–4 and 6–7 were annulled, if 5 did not occur we proceeded to 7, etc.). See Appendix for examples of two specific bacteraemic episodes.

We computed contingency tables for basic patient characteristics in relation to occurrence of all seven groups, group 1 only (most likely bacteraemia diagnosis), and the combination of groups 5, 7, 3, 4, and 6 (most likely diagnosis of or surgery for a focal infection). As basic patient characteristics we selected gender, age group (0–15, 16–64, 65–80, >80 years), Charlson comorbidity index (score 0, 1–2, >2), speciality (defining episodes as either medical, surgical, intensive care unit [ICU], paediatric, or unknown), acquisition of bacteraemia (community, healthcare-associated, nosocomial), main group of microorganisms (*Escherichia coli*, *Enterobacter* spp., *Klebsiella* spp., other Enterobacteriaceae, *Pseudomonas aeruginosa*, anaerobic Gram-negative bacteria, other Gram-negative bacteria, *Staphylococcus aureus*, coagulase-negative staphylococci [CNS], *Streptococcus pneumoniae*, haemolytic streptococci, enterococci, Gram-positive rods, other Gram-positive bacteria, fungi, polymicrobial, undetermined [0.4%]), incident vs. non-incident episodes, 30-day mortality, and, for 2,761 bacteraemic episodes (4.7%), sepsis groups (no sepsis, possibly sepsis (due to missing data), sepsis, severe sepsis/septic shock, and organ dysfunction without sepsis) [18, 20]. We used the chi-square test to assess whether characteristics differed between patients recorded with group 1–7 codes vs. no codes belonging to these groups, for group 1 codes (believed most likely to represent bacteraemia) vs. no codes belonging to this group, and for group 3–7 codes (believed most likely to represent a focal infection) vs. no codes belonging to these groups.

To assess possible time-related aspects, we depicted a histogram with the proportions of groups 1 and 3–7 on the y-axis and calendar year (2000–2011) on the x-axis.

Finally, we used logistic regression analysis to compute odds ratios (ORs) with 95% confidence intervals (CIs) for 30-day mortality, a commonly used outcome in prognostic bacteraemia studies. We adjusted for the above basic patient characteristics except sepsis groups (due to missing data). The analyses covered all bacteraemic episodes as well as subgroup analyses for groups 1–7, group 1, and groups 3–7 during 2000–2008 as data on speciality were incomplete as from 2009.

The program Stata (release 13; StataCorp) was used for all analyses.

### Ethical considerations

The study was approved by the Danish Data Protection Agency (record nos. 2007-41-0627, 2013-41-2579). Approval by an ethics committee or consent from participants (including next of kin/caregiver in the case of children) are not required for registry-based research in Denmark. Data were not anonymized prior to analysis.

## Results

We identified 58,139 bacteraemic episodes in 48,450 patients of whom 41,633 patients (85.9%) had 1 episode, 5,062 (10.5%) had 2, 1,186 (2.5%) had 3, 561 (1.2%) had 4–10, and eight had 11–16 episodes.

### Groups of diagnoses and surgical procedures

Among the 58,139 bacteraemic episodes, 37,740 (64.9%) were related to a hospitalization with one or more of the seven diagnoses/surgery groups we defined as indicative of bacteraemia or a focal infection (Table 1). Among these, 18,786 episodes (32.3%) had a group 1 code (an “infection/bacteraemia” diagnosis, i.e., the highest priority codes representing bacteraemia) with a total of 20,433 of such codes (Table 2). One such code was given to 17,309 episodes (92.1%), two codes to 1,336 episodes (7.1%) and 2–5 codes to the remaining 181 episodes (1.0%). 26,538 episodes (45.7%) had a group 3–7 code indicating a likely focal infection.

**Table 1:** Bacteraemic episodes according to groups of diagnosis/surgical procedure codes that indicate hospitalization with bacteraemia or a focal infection.

Group	No. (%) of bacteraemic episodes	Cumulative no. (%)
1) Infections/bacteraemia	18,786 (32.3)	18,786 (32.3)
2) Other diagnoses/bacteraemia	457 (0.8)	19,243 (33.1)
3) Other diagnoses/focal infection	15,110 (26.0)	34,353 (59.1)
4) Surgical procedures/focal infection	499 (0.9)	34,852 (60.0)
5) Infections/systemic infection	2,027 (3.5)	36,879 (63.4)
6) Other diagnoses/systemic infection	2 (0)	36,881 (63.4)
7) Infections/focal infection	859 (1.5)	37,740 (64.9)
None of the groups 1–7	20,399 (35.1)	58,139 (100)

**Table 2:** Group 1 (”Infections / bacteraemia”) codes, which most likely represent bacteraemia, given to 18,786 bacteraemic episodes.

Code	Text	Number (%)
DA02.1	*Salmonella* sepsis	204 (1.0)
DA28.2B	Extraintestinal yersiniosis	1 (0)
DA32.7	Listerial sepsis	43 (0.2)
DA39.2	Acute meningococcaemia	83 (0.4)
DA39.2A	Meningococcal sepsis	20 (0.1)
DA39.3	Chronic meningococcaemia	1 (0)
DA39.4	Meningococcaemia, unspecified	21 (0.1)
DA40.0	Sepsis due to streptococcus, group A	237 (1.2)
DA40.1	Sepsis due to streptococcus, group B	135 (0.7)
DA40.2	Sepsis due to streptococcus, group D	18 (0.1)
DA40.3	Sepsis due to *Streptococcus pneumoniae*	1,150 (5.6)
DA40.8	Other streptococcal sepsis	271 (1.3)
DA40.9	Streptococcal sepsis, unspecified	555 (2.7)
DA41	Other sepsis	7 (0)
DA41.0	Sepsis due to *Staphylococcus aureus*	1,611 (7.9)
DA41.1	Sepsis due to other specified staphylococcus	254 (1.2)
DA41.1A	Sepsis due to coagulase-negative staphylococcus	50 (0.2)
DA41.2	Sepsis due to unspecified staphylococcus	639 (3.1)
DA41.3	Sepsis due to *Haemophilus influenzae*	50 (0.2)
DA41.4	Sepsis due to anaerobes	386 (1.9)
DA41.5	Sepsis due to other Gram-negative organisms	5,741 (28.1)
DA41.8	Other specified sepsis	978 (4.8)
DA41.9	Sepsis, unspecified	7,224 (35.4)
DA42.7	Actinomycotic sepsis	12 (0.1)
DA49.9A	Bacteraemia, unspecified	436 (2.1)
DB37.7	Candidal sepsis	271 (1.3)
DB49.9A	Fungemia, unspecified	35 (0.2)
	Total	20,433 (100)

**Fig 1:**
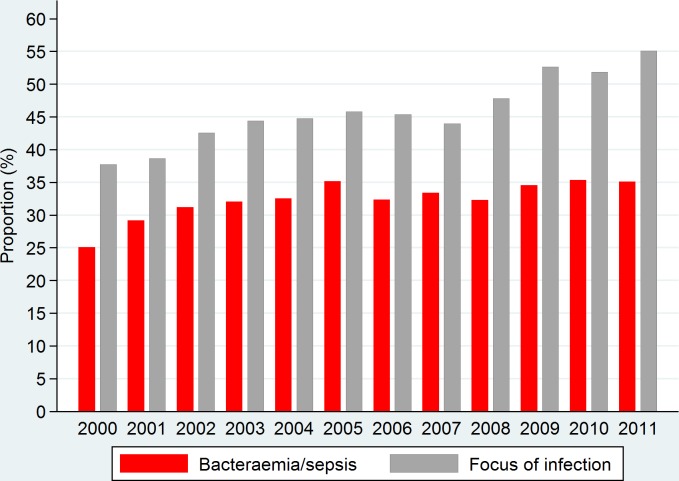
Annual proportions of bacteraemia episodes captured by ICD-10 or NOMESCO codes designating “Bacteraemia/sepsis” or “Focal infection”, 2000–2011.

For bacteraemia, an increase was detected from 2000 (25.1%) to 2005 (35.2%) after which the completeness varied between 30% and 35% (Fig 1). For infectious foci, an increase was seen from 37.7% in 2000 to 45.8% in 2005 and further from 44.0% in 2007 to 55.1% in 2011.

### Characteristics of patients with codes representing bacteraemia or a focal infection

The proportion of bacteraemic episodes that had been assigned a diagnosis/surgery group indicating bacteraemia or a focal infection (64.9%, Table 3) differed within all subgroups (p < 10^−4^) except sepsis groups (p = 0.06). Among age groups, the highest completeness was seen for the youngest (0–14 y: 70.7%) and the oldest (>80 y: 68.6%). A higher completeness was seen for females (66.6%) than for males (63.5%), patients without recorded comorbidity (71.1%), paediatric ward patients (71.8%), *E*. *coli* (73.4%), haemolytic streptococci (76.1%), non-incident bacteraemic episodes (67.2%), and no mortality within 30 days (67.7%). Pertaining to acquisition the completeness declined considerably from community (75.1%), over healthcare-associated (63.1%) to nosocomial (53.4%). For sepsis groups, the lowest completeness (61.9%) was seen for the no sepsis group, whereas higher completeness (70–71%) was encountered for the severer groups (sepsis, severe sepsis/septic shock, organ dysfunction without sepsis).

**Table 3:** Patient characteristics in relation to groups of diagnosis/surgical procedure codes that indicate hospitalization with bacteraemia or the presence of a focal infection.

Characteristic	No. episodes	No. (%) with group 1–7	No. (%) with group 1	No. (%) with group 3–7
**All**	58,139	37,740 (64.9)	18,786 (32.3)	26,538 (45.7)
**Gender**				
Females	26,848	17,883 (66.6)	8,741 (32.6)	12,793 (47.7)
Males	31,291	19,857 (63.5)	10,045 (32.1)	13,745 (43.9)
**Age group, years**				
0–14	2,112	1,493 (70.7)	624 (29.6)	763 (36.1)
15–64	19,771	12,383 (62.6)	6,026 (30.5)	8,886 (44.9)
65–80	20,257	12,892 (63.6)	6,543 (32.3)	9,163 (45.2)
>80	15,999	10,972 (68.6)	5,593 (35.0)	7,726 (48.3)
**Charlson comorbidity index score**				
0	19,684	13,988 (71.1)	5,956 (30.3)	10,587 (53.8)
1–2	22,501	14,014 (62.3)	7,294 (32.4)	9,725 (43.2)
>2	15,954	9,738 (61.0)	5,536 (34.7)	6,226 (39.0)
**Speciality** ^4^				
Medical	29,678	19,291 (65.0)	10,792 (36.4)	12,601 (42.5)
Surgical	10,652	6,110 (57.4)	1,905 (17.9)	5,091 (47.8)
Intensive care unit	3,410	1,976 (58.0)	1,100 (32.3)	1,481 (43.4)
Paediatric	1,627	1,168 (71.8)	513 (31.5)	577 (35.5)
Unknown	115	83 (72.2)	44 (38.3)	52 (45.2)
**Acquisition of bacteraemia**				
Community	25,383	19,066 (75.1)	9,192 (36.2)	13,971 (55.0)
Healthcare-associated	12,152	7,673 (63.1)	4,244 (34.9)	4,854 (39.9)
Nosocomial	20,604	11,001 (53.4)	5,350 (26.0)	7,713 (37.4)
**Group of microorganisms**				
*Escherichia coli*	16,206	11,896 (73.4)	5,895 (36.4)	8,459 (52.2)
*Enterobacter* spp.	1,232	726 (58.9)	343 (27.8)	499 (40.5)
*Klebsiella* spp.	3,893	2,468 (63.4)	1,202 (30.9)	1,711 (44.0)
Other Enterobacteriaceae	2,359	1,567 (66.4)	768 (32.6)	1,067 (45.2)
*Pseudomonas aeruginosa*	1,601	974 (60.8)	561 (35.0)	614 (38.4)
Anaerobic Gram-negative bacteria	1,361	814 (59.8)	265 (19.5)	664 (48.8)
Other Gram-negative bacteria	1,695	930 (54.9)	495 (29.2)	598 (35.3)
*Staphylococcus aureus*	7,116	4,508 (63.4)	2,605 (36.6)	2,824 (39.7)
Coagulase-negative staphylococci	2,094	960 (45.9)	424 (20.3)	676 (32.3)
*Streptococcus pneumoniae*	4,912	3,200 (65.2)	1,556 (31.7)	2,489 (50.7)
Haemolytic streptococci	2,096	1,595 (76.1)	772 (36.8)	1,110 (53.0)
Enterococci	2,916	1,865 (64.0)	883 (30.3)	1,397 (47.9)
Other Gram-positive bacteria	2,522	1,391 (55.2)	510 (20.2)	1,057 (41.9)
Gram-positive rods	1,190	619 (52.0)	283 (23.8)	412 (34.6)
Fungi	1,641	990 (60.3)	550 (33.5)	723 (44.1)
Polymicrobial	5,063	3,114 (61.5)	1,631 (32.2)	2,139 (42.3)
Unknown^5^	242	123 (50.8)	43 (17.8)	99 (40.9)
**Incident bacteraemic episode**				
Yes	48,437	31,218 (64.5)	14,976 (30.9)	22,215 (45.9)
No	9,702	6,522 (67.2)	3,810 (39.3)	4,323 (44.6)
**Sepsis group** ^6^				
No sepsis	249	154 (61.9)	73 (29.3)	110 (44.2)
Possibly sepsis	421	283 (67.2)	141 (33.5)	205 (48.7)
Sepsis	610	435 (71.3)	208 (34.1)	341 (55.9)
Severe sepsis/septic shock	1,312	921 (70.2)	581 (44.3)	616 (47.0)
Organ dysfunction, no sepsis	169	120 (71.0)	74 (43.8)	84 (49.7)
**30-day mortality**				
Yes	12,851	7,094 (55.2)	4,464 (34.7)	4,261 (33.2)
No	45,263	30,625 (67.7)	14,314 (31.6)	22,260 (49.2)
Unknown	25	21 (84.0)	8 (32.0)	17 (68.0)

^1^ Diagnosis/surgical procedure that indicate hospitalization with bacteraemia

^2^”Infections / bacteraemia” codes, which most likely represent bacteraemia, cf. Table 2

^3^ Codes, which represent the presence of a focal infection (see Appendix)

^4^ Only for bacteraemic episodes before 2009 (n = 45,482)

^5^ Mainly due to lack of speciation

^6^ Only for incident community-acquired bacteraemic episodes, Funen County, 2000–2008 (n = 2,761)

### Characteristics of patients with the most likely bacteraemia codes

The proportion of bacteraemic episodes with a group 1 code (32.3%, Table 3) differed between all subgroups (p < 10^−4^) except for gender (p = 0.24). The completeness increased with higher age groups (from 29.6% for 0–14 y to 35.0% for >80 y), Charlson comorbidity index score (from 30.3% for 0 to 34.7% for >2), and sepsis severity (from 29.3% for no sepsis to 44.3% for severe sepsis/septic shock). It was conspicuously low for surgical ward patients (17.9%) whereas a higher completeness was seen for non-incident bacteraemic episodes (39.3%) and mortality within 30 days (34.7%). Completeness for acquisition declined from community (36.2%), over healthcare-associated (34.9%) to nosocomial (26.0%). For groups of microorganisms (unknown [n = 43] excluded) the completeness varied from 19.5% for anaerobic Gram-negative bacteria to 36.8% for haemolytic streptococci.

### Characteristics of patients with the most likely focal infection codes

The proportion of bacteraemic episodes with a group 3–7 code (45.7%, Table 3) differed between all subgroups (all p < 10^−4^, except for incident vs. non-incident episodes [p = 0.02] and sepsis groups [p = 0.003]). The completeness increased with age group (from 36.1% for 0–14 y to 48.3% for >80 y) and decreased with higher Charlson comorbidity index score (from 53.8% for 0 to 39.0% for >2). A lower completeness was seen for paediatric ward patients (35.5%) and mortality within 30 days (33.2%). Completeness for acquisition declined from community (55.0%), over healthcare-associated (39.9%) to nosocomial (37.4%). For groups of microorganisms (unknown [n = 99] excluded) the completeness varied from 32.3% for CNS to 53.0% for haemolytic streptococci. Among sepsis groups, the trend of higher completeness with increasing severity was less conspicuous than for bacteraemia codes.

### 30-day mortality in multivariate analyses

The ORs (95% CIs) for 30-day mortality generally varied little whether these were computed for all bacteraemic episodes, episodes with a group 1–7 code, episodes with a group 1 code, or episodes with a group 3–7 code (Table 4) or not. However, surgical episodes with a group 1 code (OR [95% CIs]: 1.07 [0.94–1.20]), episodes with CNS (ranging from 1.24 [1.01–1.52] for episodes with a group 1–7 code to 1.43 [1.11–1.83] for episodes with a group 3–7 code), and in particular paediatric patient episodes with a group 1 code (0.98 [0.59–1.63]) or a group 3–7 code (5.32 [3.48–8.13) deviated from all the bacteraemic episodes.

**Table 4:** Odds ratios (95% confidence intervals) for 30-day mortality, adjusted for characteristics in first column, 2000–2008.

Characteristic	All episodes (n = 45,472)	With group 1–7^1^ (n = 28,620)	With group 1^2^ (n = 14,350)	With group 3–7^3^ (n = 19,796)
**Gender**				
Females	1 (ref.)	1 (ref.)	1 (ref.)	1 (ref.)
Males	0.99 (0.95–1.04)	1.01 (0.95–1.08)	1.05 (0.96–1.14)	1.01 (0.93–1.09)
**Age, per 1 year increase**	1.033 (1.032–1.035)	1.035 (1.032–1.037)	1.031 (1.028–1.034)	1.038 (1.034–1.041 =
**Charlson comorbidity index score**				
0	1 (ref.)	1 (ref.)	1 (ref.)	1 (ref.)
1–2	1.38 (1.30–1.47)	1.36 (1.26–1.47)	1.24 (1.11–1.37)	1.40 (1.27–1.55)
>2	1.95 (1.82–2.08)	1.87 (1.71–2.04)	1.68 (1.50–1.88)	1.94 (1.73–2.16)
**Speciality**				
Medical	1 (ref.)	1 (ref.)	1 (ref.)	1 (ref.)
Surgical	0.82 (0.78–0.87)	0.82 (0.75–0.89)	1.07 (0.94–1.20)	0.92 (0.83–1.02)
Intensive care unit	3.36 (3.09–3.66)	3.46 (3.09–3.88)	3.32 (2.86–3.86)	3.65 (3.18–4.20)
Paediatric	2.12 (1.64–2.74)	2.86 (2.10–3.90)	0.98 (0.59–1.63)	5.32 (3.48–8.13)
Unknown	0.78 (0.46–1.33)	0.57 (0.27–1.21)	0.52 (0.20–1.34)	0.75 (0.29–1.93)
**Acquisition of bacteraemia**				
Community	1 (ref.)	1 (ref.)	1 (ref.)	1 (ref.)
Healthcare-associated	1.33 (1.25–1.43)	1.20 (1.09–1.31)	1.12 (1.00–1.26)	1.29 (1.15–1.46)
Nosocomial	1.83 (1.72–1.94)	1.84 (1.70–1.99)	1.71 (1.54–1.89)	1.94 (1.75–2.15)
**Group of microorganisms**				
*Escherichia coli*	1 (ref.)	1 (ref.)	1 (ref.)	1 (ref.)
*Enterobacter* spp.	1.37 (1.16–1.62)	1.36 (1.08–1.73)	1.30 (0.94–1.78)	1.33 (0.97–1.82)
*Klebsiella* spp.	1.28 (1.16–1.42)	1.33 (1.16–1.53)	1.30 (1.09–1.56)	1.43 (1.19–1.71)
Other Enterobacteriaceae	1.22 (1.07–1.39)	1.36 (1.14–1.62)	1.36 (1.08–1.72)	1.58 (1.27–1.97)
*Pseudomonas aeruginosa*	1.64 (1.43–1.88)	1.80 (1.49–2.16)	1.88 (1.50–2.36)	1.89 (1.48–2.43)
Anaerobic Gram-negative bacteria	1.99 (1.72–2.32)	1.80 (1.45–2.24)	2.08 (1.51–2.88)	2.02 (1.57–2.61)
Other Gram-negative bacteria	1.35 (1.15–1.58)	1.59 (1.26–2.00)	1.81 (1.35–2.42)	1.62 (1.18–2.22)
*Staphylococcus aureus*	1.80 (1.66–1.95)	2.05 (1.85–2.28)	1.92 (1.68–2.19)	2.34 (2.03–2.69)
Coagulase-negative staphylococci	0.93 (0.81–1.07)	1.24 (1.01–1.52)	1.39 (1.05–1.85)	1.43 (1.11–1.83)
*Streptococcus pneumoniae*	1.45 (1.31–1.60)	1.79 (1.57–2.04)	1.91 (1.61–2.26)	1.96 (1.66–2.31)
Haemolytic streptococci	1.41 (1.22–1.63)	1.54 (1.29–1.84)	1.72 (1.37–2.16)	1.45 (1.14–1.85)
Enterococci	1.47 (1.31–1.64)	1.72 (1.49–2.00)	1.83 (1.50–2.22)	1.92 (1.61–2.31)
Other Gram-positive bacteria	1.25 (1.09–1.42)	1.26 (1.04–1.53)	1.37 (1.04–1.80)	1.43 (1.12–1.82)
Gram-positive rods	1.71 (1.45–2.02)	1.94 (1.53–2.46)	2.08 (1.51–2.88)	2.08 (1.53–2.82)
Fungi	2.64 (2.30–3.02)	2.59 (2.16–3.10)	2.60 (2.05–3.30)	2.70 (2.16–3.37)
Polymicrobial	2.30 (2.11–2.50)	2.29 (2.04–2.57)	2.21 (1.91–2.56)	2.36 (2.04–2.74)
Unknown^4^	0.99 (0.65–1.53)	1.11 (0.58–2.16)	0.82 (0.24–2.81)	1.21 (0.56–2.61)
**Incident bacteraemic episode**				
Yes	1 (ref.)	1 (ref.)	1 (ref.)	1 (ref.)
No	0.96 (0.90–1.02)	0.96 (0.88–1.04)	0.90 (0.81–1.00)	0.99 (0.89–1.10)

^1^ Diagnosis/surgical procedure that may indicate bacteraemia

^2^”Infections / bacteraemia” codes, which most likely represent bacteraemia, cf. Table 2

^3^ Codes that indicate a focal infection (see Appendix)

^4^ Mainly due to lack of speciation

## Discussion

Even with a comprehensive inclusion of diagnostic ICD-10 codes and NOMESCO procedure codes that could indicate either sepsis/bacteraemia or a focal infection, only 64.9% of bacteraemic episodes had at least one of these codes registered in the relevant hospital contact. With restriction to codes that more likely represented bacteraemia the completeness declined to 32.3%.

Our gold standard was bacteraemic episodes derived from positive BCs recorded in electronic laboratory information systems maintained by DCMs, from which we excluded contamination episodes by generally accepted algorithms [21, 23]. This, as well as the capture of the majority of positive BCs [17], indicates that our study database represents the greater part of detected bacteraemic episodes within well-defined geographic regions.

Bacteraemia is a serious condition [1], which should theoretically encourage its recording in administrative registries. However, many of the diagnoses that most likely capture the aetiological entity bacteraemia actually designate sepsis (Table 2). Sepsis is a clinical entity previously defined as the presence of at least two among four Systemic Inflammatory Response Syndrome (SIRS) criteria as well as infection [5, 29] and currently defined by a broader definition [30]. The correct coding of sepsis is complicated [31–36] and there is no consensus on which code abstraction strategy that will correctly capture septic episodes [37–39]. Two prior studies, a Swedish based on ICD-9 and ICD-10 codes and a US based on ICD-9 codes, compared code abstraction strategies used to retrieve severe sepsis hospitalizations from administrative registries [6, 7]. The number of severe sepsis hospitalizations varied more than three-fold, which plausibly explains the high variation when reporting incidence of sepsis [37–41].

Most administrative data validation studies either retrieved diagnostic codes from administrative registries followed by validation in randomly sampled medical charts [10, 11, 42–46] or they compared administrative registries to assess concordance [11–14, 47–49]. Fewer studies have initially scrutinized data believed to represent the gold standard followed by their completeness in administrative registries [8, 9, 50–53]

To the best of our knowledge, only the Danish ‘predecessor’ study that prompted this study has validated the diagnosis of bacteraemia in administrative registries [9]. That study included 406 bacteraemic episodes from 1994 recorded in a prospectively validated research database of positive BCs and clinical assessments [54]. Only 18 episodes (4.4%) were recorded with a bacteraemia/sepsis diagnosis in the DNPR. The DNPR replacement of ICD-8 by ICD-10 in 1994 [15] may be a reason for this low completeness. Analysis of data for our study population by using the same 30 ICD-10 codes as in the 1994 study [9] yielded a completeness of 32.0% (data not shown), which is virtually identical to the 32.3% reported here (see Appendix, group 1 codes) and thus representing a notable improvement as compared to the 4.4% reported from 1994 [9].

A few studies have validated sepsis in administrative registries, focusing on severe sepsis or septic shock [8, 50, 51]. Comparison to our study is difficult for several reasons: we do not know how much overlap there is between sepsis and bacteraemia, ICD-9 codes (which may differ substantially from ICD-10 codes [32, 55]) were used, capture of sepsis varies up to three-fold depending on the algorithm [6, 7], or the study included emergency department or ICU patients only [8, 51].

Although a low proportion of the bacteraemic episodes was recorded with a relevant diagnosis in the DNPR this may not pose a problem if this capture is non-selective, but this was not the case as proportions varied up to two-fold, e.g., 17.9% for surgical vs. 36.4% for medical patients pertaining to group 1 diagnoses. Likewise, a higher completeness was seen with increasing severity of sepsis, also reported for severe sepsis [8], whereas it declined from community over healthcare-associated to nosocomial acquisition. There were fewer variations between ORs in multivariate 30-day mortality analyses; caveat is still warranted for some subgroups though, e.g., paediatric ward patients. A meta-analysis of 36 severe sepsis trials reported the same declining mortality trend from 1993 through 2009 whether data were from the trials or administrative registries or not, though percent mortality differed considerably [56].

The Danish version of the WHO ICD-10 classification [25] is from 1993 [57] and instructions on coding and registration have not been updated since then. The codes are updated on the official Danish web site [24] and coding is facilitated by private entrepreneurs [58]. Most of the codes that cover bacteraemia include “Sepsis” in their designation, with a few exceptions, such as A49.9A [Bacteraemia, unspecified], used only for 2.1% of the Group 1 codes (Table 2). One reason for this may be that A49.9A is not included in the original code book [57], so only physicians who are aware of the web amendments [24, 58] will probably use this highly relevant code. A future update could alter the designations in the Group 1 codes from “Sepsis” to “Bacteraemia”, as this would be more in accordance with globally accepted definitions [5] and would probably facilitate and increase the recording of bacteraemia in the DNPR.

We used a database which represented clinically important bacteraemic episodes, the study was population-based, and included a high number of episodes that enabled subgroup analyses. However, there were also limitations that warrant further discussion. The main limitation was the inability to report predictive values as we had no information on the use of the 1,079 codes in the DNPR for patients not having bacteraemia. In the Danish ‘predecessor’ study, codes that roughly correspond to our group 1 codes were assessed for all patients in the DNPR, which enabled the reporting of positive predictive values (PPVs), found to be 21.7% [9]. PPVs would probably decline with the inclusion of group 1–7 or group 3–7 codes, in which the prevalence of bacteraemia would not alter the numerator, but the denominator comprising all the said codes would increase. Such low PPVs, and consequently many false positive patients, preclude research on bacteraemia patients based on administrative data. Secondly, we only had clinical data for 4.7% of the bacteraemic episodes. The increasing completeness with higher sepsis severity further indicated the selective recording of patient groups in administrative registries. Thirdly, the multiple ICD-10 codes prompted us to define a limited number of groups based on the likelihood to represent bacteraemia. Although we used a consensus process the classification of codes and the ranking of groups were subjective. Still, the ordinal scale provided a working solution to the conundrum of 5,040 ordered sequences of the 7 groups. Finally, some blood cultures with common skin commensals may represent contamination, and not bacteraemia, which may be reflected in the lower completeness for CNS (Table 3). However, for 9,482 bacteraemic episodes (part of the actual study database) we previously reported 94.6% agreement for bacteraemia vs. contamination when computer algorithms were compared to physicians’ individual clinical assessments [21]. We therefore believe this had minor impact on the results.

In conclusion, our study showed a low completeness of bacteraemic episodes identified in the official Danish administrative registry used for the recording of all hospital contacts. Further, there were considerable differences in completeness as to whether the acquisition was community, healthcare-associated or nosocomial. Although few studies have shown this for bacteraemia, this is in accordance with sepsis [33, 35] and health-care associated infections [55, 59] for which it has been concluded that their detection should not be based solely on administrative registry data. Hence, bacteraemia studies should preferably be derived from bacteraemia databases based on positive blood cultures [54, 60, 61].

## Appendix

### Diagnosis/operation groups, their codes, and text examples of the three most common codes within each group, followed by two examples of prioritization

*Group 1: Infections/bacteraemia*

DA02.1, DA20.7, DA21.7, DA22.7, DA22.9B, DA26.7, DA28.2B, DA32.7, DA39.2, DA39.2A, DA39.3, DA39.4, DA40, DA40.0, DA40.1, DA40.2, DA40.3, DA40.8, DA40.9, DA41, DA41.0, DA41.1, DA41.1A, DA41.2, DA41.3, DA41.4, DA41.5, DA41.8, DA41.9, DA41.9C, DA42.7, DA49.9A, DA54.8G, DB37.7, DB49.9A

The three most common diagnoses from Group 1: See article, Table 2

*Group 2: Other diagnoses/bacteraemia*

DO75.3A, DO85.9, DP36, DP36.0, DP36.1, DP36.2, DP36.3, DP36.4, DP36.5, DP36.8, DP36.9

The three most common diagnoses from Group 2 (no./all group codes [%]):

DP36.9 –Bacterial sepsis of newborn, unspecified (2,749/3,816 [72.0])

DP36.2 –Sepsis of newborn due to *Staphylococcus aureus* (904/3,816 [23.7])

DP36.0—Sepsis of newborn due to streptococcus, group B (80/3,816 [2,1])

*Group 3: Other diagnoses/focal infection*

DD73.3, DE06.0A, DE06.0B, DE10.5C, DE11.5C, DE12.5C, DE13.5C, DE13.5D, DE14.5B, DE14.5C, DE23.6A, DE32.1, DG00, DG00.0, DG00.1, DG00.2, DG00.3, DG00.8, DG00.8A, DG00.8B, DG00.9, DG00.9A, DG01, DG01.9, DG01.9B, DG01.9C, DG01.9D, DG01.9G, DG01.9H, DG01.9I, DG02, DG02.1A, DG02.1C, DG02.8, DG04.2, DG04.2A, DG04.2B, DG05.0, DG05.0E, DG05.0H, DG05.0I, DG05.0O, DG05.0P, DG05.0S, DG05.2, DG05.2L, DG05.2M, DG05.2N, DG06, DG06.0, DG06.0A, DG06.0B, DG06.0C, DG06.0D, DG06.0E, DG06.0F, DG06.1, DG06.1A, DG06.1B, DG06.1C, DG06.2, DG06.2A, DG06.2B, DG06.2C, DG07, DG07.9, DG07.9A, DG07.9B, DG07.9C, DG07.9E, DG08.9A, DG08.9B, DG08.9C, DG08.9D, DG08.9E, DG08.9F, DG08.9G, DG08.9H, DG08.9I, DG08.9J, DG08.9K, DG08.9L, DG08.9M, DG08.9N, DH05.0, DH05.0A, DH05.0B, DH05.0E, DH44.0, DH44.0B, DH70.0, DH70.0A, DH70.0B, DH70.1, DH75.0, DH75.0B, DI30.1, DI30.1A, DI30.1B, DI30.1C, DI30.1D, DI32.0, DI32.0A, DI32.0B, DI32.0C, DI32.1, DI32.1A, DI32.1B, DI32.8A, DI33, DI33.0, DI33.0A, DI33.0B, DI33.0C, DI33.0D, DI33.0E, DI33.0F, DI33.9, DI38, DI39.0, DI39.1, DI39.2, DI39.3, DI39.4, DI39.8, DI39.8A, DI39.8B, DI39.8D, DI39.8E, DI40, DI40.0, DI40.0A, DI41.0, DI41.0B, DI41.0C, DI52.0, DI52.0A, DI68.1, DI68.1A, DJ01.0A, DJ01.0B, DJ01.1A, DJ01.1B, DJ01.2A, DJ01.2B, DJ01.3A, DJ01.3B, DJ01.4A, DJ01.4B, DJ02.9C, DJ03.9D, DJ13, DJ13.9, DJ13.9A, DJ13.9B, DJ14, DJ14.9, DJ14.9A, DJ14.9B, DJ15, DJ15.0, DJ15.1, DJ15.2, DJ15.3, DJ15.4, DJ15.5, DJ15.6, DJ15.6A, DJ15.7, DJ15.8, DJ15.9, DJ16, DJ17.0, DJ17.0A, DJ17.0B, DJ17.0C, DJ17.0D, DJ17.0E, DJ17.0F, DJ17.0G, DJ17.1, DJ18.8, DJ34.0, DJ36.9, DJ39.0, DJ39.0A, DJ39.0B, DJ39.0C, DJ39.1A, DJ85, DJ85.0, DJ85.0A, DJ85.1, DJ85.2, DJ85.3, DJ86, DJ86.0, DJ86.0A, DJ86.9, DJ86.9A, DJ95.0A, DJ98.5D, DK04.1A, DK11.3, DK11.3A, DK11.3B, DK11.3C, DK11.3D, DK12.2, DK12.2A, DK12.2B, DK13.0A, DK14.0A, DK20.9A, DK35, DK35.0, DK35.0A, DK35.1, DK35.1A, DK35.2, DK35.3, DK35.3A, DK35.3B, DK35.8, DK35.8A, DK35.8B, DK35.8C, DK35.9, DK35.9A, DK35.9B, DK36, DK40.1, DK40.4, DK41.1, DK41.4, DK42.1, DK43.1, DK43.4, DK43.7, DK44.1, DK45.1, DK45.1B, DK45.1C, DK45.1D, DK45.1E, DK45.1F, DK45.1G, DK45.1H, DK45.1I, DK45.1J, DK45.1K, DK45.1L, DK45.1M, DK46.1, DK55.0A, DK55.0B, DK55.0C, DK55.0D, DK55.1B, DK55.1C, DK55.1D, DK57, DK57.0, DK57.0A, DK57.0B, DK57.0C, DK57.1, DK57.2, DK57.2A, DK57.2B, DK57.2C, DK57.3, DK57.4, DK57.4A, DK57.5, DK57.8, DK57.9, DK57.9A, DK59.3C, DK61.0, DK61.0A, DK61.0B, DK61.1, DK61.1A, DK61.2, DK61.3, DK61.4, DK63.0, DK63.8A, DK65, DK65.0, DK65.0A, DK65.0B, DK65.0C, DK65.0D, DK65.0E, DK65.0F, DK65.0G, DK65.0H, DK65.0I, DK65.0J, DK65.0K, DK65.0L, DK65.0M, DK65.0N, DK65.0O, DK65.0P, DK65.8, DK65.8I, DK67.1, DK75.0, DK75.0A, DK75.0B, DK75.0C, DK75.0D, DK80.0, DK80.0A, DK80.0B, DK80.0C, DK80.0D, DK80.1, DK80.1C, DK80.3, DK80.3A, DK80.3B, DK80.3C, DK80.4, DK80.4A, DK80.4B, DK80.4C, DK80.4D, DK80.4E, DK81.0, DK81.0A, DK81.0B, DK81.0C, DK81.0D, DK81.1, DK83.0, DK83.0A, DK83.0B, DK83.0C, DK83.0D, DK83.0E, DK85, DK85.0, DK85.1, DK85.1A, DK85.8A, DK85.8E, DK85.9, DK85.9A, DK85.9B, DK85.9D, DK85.9E, DK86.1A, DL02.0, DL02.1B, DL02.1C, DL02.2G, DL02.2H, DL02.2I, DL02.2J, DL02.2K, DL02.2L, DL02.2M, DL02.2N, DL02.2R, DL02.2S, DL02.2T, DL02.8A, DL02.8B, DL02.8C, DL03.0A, DL03.0F, DL03.1, DL03.1A, DL03.1B, DL03.1C, DL03.1D, DL03.1E, DL03.1F, DL03.1G, DL03.1H, DL03.1I, DL03.3, DL03.3A, DL03.3B, DL03.3C, DL03.3D, DL03.3E, DL03.3F, DL03.8, DL03.8A, DL08.0, DL08.0A, DL08.0a, DL08.8B, DM00, DM00.0, DM00.0A, DM00.0B, DM00.1, DM00.1A, DM00.1B, DM00.2, DM00.2A, DM00.2B, DM00.8, DM00.9, DM01, DM01.0, DM01.1, DM01.3, DM01.3A, DM01.3B, DM01.3C, DM01.6, DM46.2, DM46.3, DM46.3A, DM46.4, DM46.5, DM46.5A, DM49.0, DM49.1, DM49.2, DM49.3, DM49.3A, DM60.0, DM60.0A, DM60.8, DM60.8A, DM60.8A1, DM60.9, DM63.0, DM65.0, DM65.1, DM68.0, DM68.0G, DM68.0H, DM72.5A, DM72.6, DM86, DM86.0, DM86.1, DM86.2, DM86.3, DM86.4, DM86.5, DM86.5A, DM86.6, DM86.8, DM86.8A, DM86.9, DM86.9A, DM90.2, DM90.2C, DN10, DN10.9, DN10.9A, DN10.9B, DN10.9C, DN11, DN11.0, DN11.0A, DN11.1, DN11.1A, DN11.1B, DN11.2, DN11.8, DN12, DN12.9, DN13.6, DN13.6A, DN13.6B, DN13.6C, DN13.6D, DN13.6E, DN15.1, DN15.1A, DN15.1B, DN20.9A, DN30, DN30.0, DN30.1, DN30.8A, DN34.0, DN39.0, DN41.0, DN41.1, DN41.2, DN41.2A, DN41.3, DN41.3A, DN41.8, DN45.0, DN45.0A, DN45.0B, DN45.0C, DN45.0D, DN45.9, DN48.2A, DN48.2B, DN48.2C, DN48.2D, DN48.2E, DN48.2F, DN48.2H, DN48.2I, DN49.8C, DN51.0A, DN51.1E, DN51.1H, DN61.9B, DN61.9E, DN61.9F, DN70.0B, DN70.0C, DN70.0D, DN70.0F, DN70.0G, DN70.1, DN70.1A, DN71.0, DN71.0A, DN71.0B, DN71.0C, DN71.0D, DN71.0E, DN71.0F, DN73.0, DN73.0A, DN73.0B, DN73.0C, DN73.0D, DN73.0E, DN73.1A, DN73.1B, DN73.2A, DN73.2B, DN73.3, DN73.3A, DN73.4A, DN73.5A, DN73.8A, DN73.8B, DN73.8C, DN75.1, DN75.8A, DN76.0A, DN76.0B, DO03.0, DO03.5, DO03.5A, DO03.5B, DO04.0, DO04.5, DO04.5A, DO04.5B, DO07.0, DO07.5, DO08.0, DO08.0A, DO08.0B, DO08.0C, DO08.0D, DO08.0E, DO08.0F, DO08.0G, DO08.0H, DO08.0I, DO08.0J, DO08.0K, DO08.0L, DO08.0M, DO08.0N, DO08.0O, DO08.0P, DO08.0Q, DO08.0R, DO23, DO23.0, DO41.1, DO41.1A, DO41.1B, DO41.1C, DO41.1D, DO75.2, DO75.3, DO85, DO85.9A, DO85.9B, DO86, DO86.0, DO86.0A, DO86.0B, DO86.0C, DO86.0D, DO86.1C, DO86.2, DO86.2B, DO86.3, DO88.3, DO91.1, DO91.1D, DO91.1E, DO91.1F, DO91.1G, DO91.1H, DO91.1I, DO91.1J, DO91.1K, DO91.1L, DO91.2B, DO91.2C, DO91.2D, DO91.2E, DP02.7, DP02.7A, DP02.7B, DP02.7C, DP15.4A, DP23, DP23.2, DP23.3, DP23.4, DP23.5, DP23.6, DP23.6A, DP23.6B, DP23.6C, DP38.9, DP38.9A, DP38.9B, DP38.9C, DP38.9D, DP39.2, DP39.3, DP39.4, DP39.8, DP39.9, DP58.2, DP77, DP77.9, DT79.3, DT80.1A, DT80.1B, DT80.1C, DT80.2, DT80.2A, DT80.2B, DT80.2C, DT80.2D, DT80.2E, DT80.2F, DT81.4, DT81.4A, DT81.4B, DT81.4C, DT81.4D, DT81.4F, DT81.4G, DT81.4H, DT81.4I, DT81.4J, DT81.4P, DT81.4U, DT81.4X, DT82.6, DT82.6A, DT82.7, DT82.7A, DT82.7B, DT82.7I, DT82.7P, DT83.5A, DT83.5B, DT83.5C, DT83.6, DT83.6A, DT83.6B, DT83.6C, DT84.5, DT84.5A, DT84.6, DT84.6A, DT84.7, DT85.7, DT87.4, DT88.0, DT89

The three most common diagnoses from Group 3 (no./all group codes [%]):

DN30.0—Acute cystitis (1,025/1,936 [52.9])

DN39.0—Urinary tract infection, site not specified (410/1,936 [21.2])

DN10.9—Acute tubulo-interstitial nephritis, unspecified (114/1,936 [5.9])

*Group 4: Surgical procedures/focal infection*

KAAM, KAAM00, KAAM10, KAAM99, KAWC, KAWC00, KAWC00A, KBWC, KBWC00, KFWC, KFWC00, KFXA10, KGWC, KGWC00, KGWC01, KHWC, KHWC00, KJAJ, KJAJ00, KJJA10A, KJLD13, KJLD13C, KJLD13D, KJWC, KJWC00, KJWC01, KKWC, KKWC00, KKWC01, KLWC, KLWC00, KLWC01, KMWC, KMWC00, KMWC01, KNAS, KNAS1, KNAS10, KNAS11, KNAS12, KNAS13, KNAS14, KNAS15, KNAS16, KNAS19, KNAS2, KNAS20, KNAS21, KNAS22, KNAS23, KNAS24, KNAS25, KNAS26, KNAS29, KNAS4, KNAS40, KNAS41, KNAS42, KNAS43, KNAS44, KNAS45, KNAS46, KNAS49, KNAS5, KNAS50, KNAS51, KNAS52, KNAS53, KNAS54, KNAS55, KNAS56, KNAS59, KNAS6, KNAS60, KNAS61, KNAS62, KNAS63, KNAS64, KNAS65, KNAS66, KNAS69, KNAS9, KNAS90, KNAS91, KNAS92, KNAS93, KNAS94, KNAS95, KNAS96, KNAS99, KNAU89, KNAW69, KNBS, KNBS09, KNBS19, KNBS29, KNBS39, KNBS49, KNBS59, KNBS99, KNBU89, KNBU89A, KNBU89B, KNBW69, KNCS, KNCS09, KNCS19, KNCS29, KNCS39, KNCS49, KNCS59, KNCS99, KNCU89, KNCU89A, KNCU89B, KNCW69, KNDS, KNDS09, KNDS19, KNDS29, KNDS39, KNDS49, KNDS59, KNDS99, KNDU89, KNDU89A, KNDU89B, KNDW69, KNES, KNES19, KNES29, KNES49, KNES59, KNES99, KNEU89, KNEW69, KNFS, KNFS19, KNFS29, KNFS49, KNFS59, KNFS99, KNFU89, KNFU89A, KNFU89B, KNFW69, KNGS, KNGS09, KNGS19, KNGS29, KNGS39, KNGS49, KNGS59, KNGS99, KNGU89, KNGU89A, KNGU89B, KNGW69, KNHS, KNHS09, KNHS19, KNHS29, KNHS39, KNHS49, KNHS59, KNHS99, KNHU89, KNHU89A, KNHU89B, KNHW69, KPJW10, KPWC, KPWC00, KQWB, KQWB00, KQWB10, KQWC, KQWC10, KTJA40, KTJL10, KTKA20

The three most common procedures from Group 4 (no./all group codes [%]):

KTJA40—Percutaneous local drainage of peritoneal cavity (204/538 [37.9])

KJWC00—Reoperation for deep infection in gastroenterological surgery (107/538 [19.9])

KTJL10—Percutaneous drainage of pseudocyst or abscess of pancreas (77/538 [14.3])

*Group 5: Infections/systemic infection*

DA01, DA01.0, DA01.1, DA01.2, DA01.3, DA01.4, DA20, DA20.9, DA21, DA21.9, DA22, DA22.9, DA23, DA23.0, DA23.1, DA23.2, DA23.3, DA23.8, DA23.9, DA23.9A, DA23.9B, DA23.9C, DA23.9D, DA24, DA24.0, DA24.0A, DA24.1, DA24.2, DA24.3, DA24.4, DA25, DA25.0, DA25.0A, DA25.1, DA25.9, DA26, DA26.9, DA27, DA27.0, DA27.8, DA27.8A, DA27.8B, DA27.8C, DA27.8D, DA27.9, DA28.0, DA32, DA32.8A, DA39, DA39.1, DA41.8, DA41.9, DA41.9A, DA41.9B, DA42.7, DA43.9, DA44.0, DA44.1, DA44.8, DA48.4A, DA49, DA49.1, DB37, DB38.7, DB39.3, DB40.7, DB41.1, DB42.7, DB44.7, DB45, DB45.7, DB46.4, DB95, DB96, DB98.1

The three most common diagnoses from Group 5 (no./all group codes [%]):

DA41.9B –Urosepsis (12/15 [80.0])

DA41.9A –Septic shock (2/15 [13.3])

DA01.0 –Typhus (1/15 [6.7])

*Group 6: Other diagnoses/systemic infection*

DJ02.0A, DO08.0S, DO08.0T, DO08.0U, DO08.0V, DO08.0X, DO08.0Y, DO08.2J, DO08.2K, DO08.2L, DP36.9, DP37.2, DP37.5

The three most common diagnoses from Group 6 (no./all group codes [%]):

DP37.5—Neonatal candidiasis (3,833/27,976 [13.7])

DO08.0U –Sepsis after abortion (2,949/27,976 [10.5])

DO08.0V –Septic shock after molar pregnancy (1,776/27,976 [6.3])

*Group 7: Infections/focal infection*

DA02, DA02.0, DA02.2, DA02.2A, DA02.2B, DA02.2C, DA02.2D, DA02.2E, DA02.8, DA04.5, DA04.6, DA04.7, DA04.8, DA04.9, DA20.0, DA20.1, DA20.2, DA20.3, DA20.8, DA21.0, DA21.1, DA21.2, DA21.3, DA21.8, DA22.0, DA22.1, DA22.2, DA22.8, DA22.9A, DA22.9C, DA22.9D, DA26.0, DA26.8, DA26.9, DA28.2, DA28.2A, DA32.0, DA32.1, DA32.1A, DA32.1B, DA32.8, DA32.9, DA39.0, DA39.5, DA39.5A, DA39.5B, DA39.8, DA42.8, DA43, DA43.0, DA43.1, DA43.8, DA46, DA46.9, DA48.0, DA54.4, DA54.4A, DA54.4B, DA54.4C, DA54.4D, DA54.8A, DA54.8C, DA54.8D, DA54.8E, DA54.8F, DA69.0C, DA69.1, DA69.1G, DB37.1, DB37.6, DB45.1, DB45.1A, DB45.1B, DI38.9, DI39, DT80.2D1, DT80.2G

The three most common diagnoses from Group 7 (no./all group codes [%]):

DA46.9 –Erysipelas, unspecified (843/2,272 [37.1])

DI38.9 –Endocarditis, unspecified (287/2,272 [12.6])

DA39.0—Meningococcal meningitis (190/2,272 [8.4])

Example 1:

A bacteraemic episode with the following 4 codes recorded:

DA41.0—Sepsis due to *Staphylococcus aureus* (Group 1)

DA41.9A –Septic shock (Group 2)

DK85.9—Acute pancreatitis, unspecified (Group 6)

KTJL10—Percutaneous drainage of pseudocyst or abscess of pancreas (Group 7)

With a group prioritization of 1 > 2 > 3 > 4 > 5 > 6 > 7, this bacteraemic episode will be recorded as DA41.0—Sepsis due to *Staphylococcus aureus* (Group 1), annulling the three other codes.

With a group prioritization of 5 > 7 > 3 > 4 > 6 > 1 > 2, the same bacteraemic episode will be recorded as KTJL10—Percutaneous drainage of pseudocyst or abscess of pancreas (Group 7), annulling the three other codes.

Example 2:

A bacteraemic episode with the following 3 codes recorded:

DA41.9B –Urosepsis (Group 2)

DP364—Sepsis of newborn due to *Escherichia coli* (Group 4)

DG008B - Meningitis from *Escherichia coli* (Group 6)

With a group prioritization of 1 > 2 > 3 > 4 > 5 > 6 > 7, this bacteraemic episode will be recorded as DA41.9B –Urosepsis (Group 2), annulling the two other codes.

With a group prioritization of 5 > 7 > 3 > 4 > 6 > 1 > 2, the same bacteraemic episode will be recorded as DP364—Sepsis of newborn due to *Escherichia coli* (Group 4), annulling the two other codes.
